# Combined Loop-Mediated Isothermal Amplification Assays for Rapid Detection and One-Step Differentiation of *Campylobacter jejuni* and *Campylobacter coli* in Meat Products

**DOI:** 10.3389/fmicb.2021.668824

**Published:** 2021-06-09

**Authors:** Antonia Kreitlow, André Becker, Marwa F. E. Ahmed, Sophie Kittler, Ulrich Schotte, Madeleine Plötz, Amir Abdulmawjood

**Affiliations:** ^1^Institute for Food Quality and Food Safety, University of Veterinary Medicine Hannover, Hanover, Germany; ^2^Institute for Animal Hygiene, Animal Welfare and Farm Animal Behavior, University of Veterinary Medicine Hannover, Hanover, Germany; ^3^Department of Hygiene and Zoonoses, Faculty of Veterinary Medicine, Mansoura University, Mansoura, Egypt; ^4^Department A-Veterinary Medicine, Central Institute of the Bundeswehr Medical Service Kiel, Kronshagen, Germany

**Keywords:** LAMP assay, *rplD* gene, *cdtC* gene, *gyrA* gene, *Campylobacter*, meat products

## Abstract

A loop-mediated isothermal amplification (LAMP) assay system was established, allowing *rplD* gene-based simultaneous detection of *Campylobacter jejuni* and *Campylobacter coli* in enriched meat products. Additionally, one-step differentiation of target species on agar plates was enabled by *cdtC* gene- and *gyrA* gene-based duplex LAMP. Both the *rplD* and *cdtC*–*gyrA* LAMP assays amplified the target sequences in all 62 *C. jejuni* and 27 *C. coli* strains used for determining inclusivity and revealed 100% exclusivity toward 85 tested non-target species. Throughout the entire experiments, *C. jejuni* and *C. coli* strains were 100% distinguishable by melting curves of *cdtC* and *gyrA* LAMP products. After 24-h enrichment, the *rplD* LAMP assay reliably detected initial inoculation levels of 10–100 CFU/g in artificially contaminated minced meat. Investigation of naturally contaminated meat samples revealed a diagnostic accuracy of 95% toward real-time PCR and 94.1% toward the standard culture method applying the 24-h incubation period. Diagnostic sensitivity and specificity, and positive and negative predictive values were 89.8, 100, 100, and 91.2%, respectively, when measured against real-time PCR, and 89.6, 98.1, 97.7, and 91.2%, respectively, when measured against the standard culture method. After 48-h enrichment, the detection limit of the *rplD* LAMP assay improved to initial inoculation levels of 1–10 CFU/g in artificially contaminated minced meat. Applying the 48-h incubation period on naturally contaminated meat samples resulted in 100% concordant results between *rplD* LAMP, real-time PCR, and the standard culture method. The established LAMP assay system was proved to be suitable for rapid meat sample screening. Furthermore, it constitutes a promising tool for investigating other *Campylobacter* sources and could therefore make a valuable contribution to protect consumers from foodborne illness.

## Introduction

*Campylobacter* infections occur in countries worldwide and have moderate impact on public health and economy. Particularly high numbers of associated disability-adjusted life years (DALY) are accounted for African and Southeast Asian regions (Devleesschauwer et al., 2017). In 2018, campylobacteriosis represented the most frequently reported foodborne zoonosis in the European Union. Status tracking of infected persons revealed that hospitalization was necessary for 30.6% of the registered cases, reflecting the economic significance of the disease (European Food Safety Authority and European Centre for Disease Prevention and Control, 2019). Besides the classic gastrointestinal disorders of variable severity, campylobacteriosis can contribute to serious gastrointestinal and extraintestinal sequelae, such as inflammatory bowel diseases and colorectal cancer or Guillain–Barré syndrome and reactive arthritis (Kaakoush et al., 2015). Several investigations revealed that species *Campylobacter jejuni* and *Campylobacter coli* were involved in most of the confirmed human campylobacteriosis cases (Skarp et al., 2016; European Food Safety Authority and European Centre for Disease Prevention and Control, 2019). Broiler meat constitutes a major source of transmission, with infections often occurring as a result of cross contamination during handling or, less frequently, the consumption of undercooked meat products (Nauta et al., 2009). Rapid screening methods for detecting the fastidious *Campylobacter* species in food are widely missing but could help to protect people from foodborne illness. As a preventive tool for fast hazard identification, these contribute to increased risk awareness, having a significant impact on responsible food handling by consumers (Her et al., 2020). This might also be a valuable aspect for an improvement in industrial hygiene and the associated reduction of *Campylobacter* contamination in retail products. In this regard, the significance of the standard culture method in accordance with DIN EN ISO 10272-1:2017 (International Organization for Standardization, 2017) is limited since investigations for the presence of *Campylobacter* spp. in fresh meat products take a minimum of 4–5 days. In contrast, nucleic acid amplification-based techniques provide much faster results. Some polymerase chain reaction (PCR) assays have been developed, enabling detection and differentiation of *C. jejuni* and *C. coli* in food samples after 16- to 48-h enrichment (Mateo et al., 2005; Mayr et al., 2010; Saiyudthong et al., 2015). However, performing conventional PCR is still time-consuming and prone to contamination, while real-time PCR systems require expensive laboratory equipment without any opportunities for possible on-site applications. Since 2000, the loop-mediated isothermal amplification (LAMP) method has gained increased attention, as it offers several advantages over PCR. Reactions take place under isothermal conditions without the necessity for a thermal cycler device. DNA amplification can be performed using a heating block and monitored with the naked eye, e.g., by turbidity or fluorescence observation (Mori and Notomi, 2009; Mori et al., 2013). Additionally, it was shown that the LAMP technique is robust against potential reaction inhibitors and copes with simplified DNA extraction procedures (Kaneko et al., 2007; Kreitlow et al., 2021). These characteristics make the LAMP method suitable for low-cost and rapid detection of pathogens with options for on-site application or implementation under restricted laboratory conditions often found in developing countries. Most of the previously established *Campylobacter* LAMP assays focused on the detection of *C. jejuni* and *C. coli* in primary production or at post-harvest level (Dong et al., 2014; Sabike et al., 2016; Romero and Cook, 2018). In addition, there are some assays for diagnostic applications in humans that allow direct detection of these pathogens in feces (Ushijima et al., 2014; Pham et al., 2015). Very few approaches have been made using LAMP for detecting *C. jejuni* and *C. coli* in food (Yamazaki et al., 2009; Zang et al., 2017). Unlike clinical specimen and samples from primary production or carcasses, retail samples undergo processing, packaging, and storage. Due to the low tenacity of *Campylobacter* spp., this might affect the survival, viability, and culturability of the bacterial cells and could therefore have an impact on pathogen detectability (Byrd et al., 2011; Duqué et al., 2019). However, this effect was not evaluated in previous studies about LAMP-based detection of *C. jejuni* and *C. coli* in meat samples. In the present study, combined LAMP assays were developed and validated for the simultaneous detection of *C. jejuni* and *C. coli* in retail meat products and subsequent species differentiation directly from agar plates. One aim of the study was to gain a better understanding of the extent to which impaired *Campylobacter* fitness adversely affects the detection capability of LAMP and how the LAMP system compares with a cultural and alternative molecular diagnostic method. The LAMP assay for simultaneous detection of both species targeted gene *rplD*, while target genes *cdtC* and *gyrA* were used for species differentiation between *C. jejuni* and *C. coli*, respectively. To the best of our knowledge, this is the first study on *rplD*-based detection and *cdtC*- and *gyrA*-based differentiation of *C. jejuni* and *C. coli* by LAMP.

## Materials and Methods

### LAMP Primer Design

Three LAMP primer sets targeting the *rplD* gene, universally present in both *C. jejuni* and *C. coli*, as well as the *C. jejuni*-specific *cdtC* gene and the *C. coli*-specific *gyrA* gene were designed via LAMP Designer software 1.15 (PREMIER Biosoft, San Francisco, CA, United States; Table 1). Corresponding nucleotide sequence data were obtained from the National Center for Biotechnology Information (NCBI) database GenBank (Bethesda, MD, United States). Sequences of the *rplD* and *cdtC* gene originated from *C. jejuni* NCTC 12660 (GenBank accession no. CP028910.1), while the sequence of the *gyrA* gene was derived from *C. coli* MG 1116 (GenBank accession no. CP017868.1). Target gene specificity was verified using the nucleotide Basic Local Alignment Search Tool (BLAST) algorithm by NCBI. Therefore, local alignments of *rplD*, *cdtC*, and *gyrA* sequences were generated against other *C. jejuni* and *C. coli* genomes as well as genomes of other *Campylobacter* and non-*Campylobacter* species available at the GenBank database. For evaluating target gene suitability, resulting similarities among target species were assessed under consideration of single-nucleotide polymorphisms. Primer sets proposed by the LAMP designer software, consisting of forward and backward outer primers (F3/B3), forward and backward inner primers (FIP/BIP), and forward and backward loop primers (LF/LB), were selected by ranking and checked for their location in conserved gene regions. The order of the corresponding oligonucleotides in HPSF-purified quality was placed with Eurofins Genomics GmbH (Ebersberg, Germany).

**TABLE 1:** *RplD*-, *cdtC*-, and *gyrA*-based primer sequences used for LAMP assay establishment.

Primer	Sequence (5′–3′)	Position
***rplD* (GenBank accession no. CP028910)**		
F3	GTGATGTAAGTGGTGGTGG	1662231–1662249
B3	AAGCATCTTTGACGCCAA	1661956–1661973
FIP (F1c + F2)	TTTGTTGGACCAAAAGCAACCG-CTAGAGCGGGTTCAACAAG	1662132–1662153/1662195–1662177
BIP (B1c + B2)	AAAGATTGGCGCTTGAAAGAGC-CAATAGCCAAAGAATCAGCAG	1662072–1662093/1662016–1662036
LF	CGCCTACCCAAACGTTAGTT	1662157–1662176
LB	GCAGATAAAGCAGCTAAAGGTG	1662067–1662046
***cdtC* (GenBank accession no. CP028910)**		
F3	GGGTAGCAGCTGTTAAAGG	89252–89270
B3	CGCCTTTAGGGATACCTCA	89667–89685
FIP (F1c + F2)	AATGCCAACAACAACTTCAG-GGCTATTCCAAAGCGTTT	89426–89445/89331–89348
BIP (B1c + B2)	TGCTCCAAAGGTTCCATCTTCT-TAGCTGATGAACTTCCTT	89499–89520/89576–89593
LF	CTGTGCAAATTCGTTCTTTAG	89405–89425
LB	TGGCTAAACAAAGATCGCTTTC	89525–89546
***gyrA* (GenBank accession no. NZ_CP017868)**		
F3	CCTTAGTAAGAATGGCACAAGA	830429–830450
B3	CTAGCATCCTTATGATCAAGCA	830756–830777
FIP (F1c + F2)	CAGTATAACGCATTGCAGCAGC-TCTATGCGTTATCCAAGTATCG	830518–830536/830455–830476
BIP (B1c + B2)	ACGATGATTCTATGAGTGAGCC-CTCGTTAAGACTATGCGGAG	830612–830633/830714–830733
LF	CCATCACCATCGATAGAACCAA	830492–830511
LB	TATTGCTGTAGGTATGGCGAC	830685–830705

### LAMP Assay Optimization

For optimal adjustment of assay parameters, LAMP reaction performance was evaluated under the application of different reaction temperatures and primer concentrations. All LAMP reactions were carried out using the real-time fluorometer Genie^®^ II (OptiGene Ltd., Horsham, United Kingdom). The device weighs about 2 kg, is portable, and includes a rechargeable battery for potential on-site use. It is equipped with two heating blocks offering eight sample positions each and can be operated via a touchscreen. Amplification and melting curve generation is directly observable on the device. While detection times and melting temperatures are displayed on the instrument, detailed analysis can be performed using the application software Genie Explorer (OptiGene Ltd.). In the first step, primer mixes of the three LAMP primer sets were prepared in a standard and concentrated version in accordance with the recommendations of OptiGene Ltd. Thus, in a LAMP reaction mixture, the concentration of each outer, inner, and loop primers was 0.2, 0.8, and 0.4 μM, respectively, using the standard primer mix, and 0.2, 2, and 1 μM, respectively, using the concentrated primer mix. In a second step, both the standard and concentrated primer mixes of each primer set were tested at temperatures ranging from 62 to 69°C. The corresponding temperature gradient (Δ = 1°C) was set over the eight wells of a heating block at the Genie^®^ II device. Each LAMP reaction mixture with a total volume of 25 μl contained 15 μl of GspSSD isothermal master mix (ISO-001) (OptiGene Ltd.), 2.5 μl of primer mix, 2.5 μl of nuclease-free water (Qiagen GmbH, Hilden, Germany), and 5 μl of DNA template (0.1 ng/μl). Adjustment of assay parameters for *rplD* and *cdtC* primers was based on DNA of *C. jejuni* NCTC 12660, while DNA of *C. coli* NCTC 12668 served as the template for *gyrA* primer-specific adjustment. After a 40-min isothermal amplification period, the reaction was terminated by heating to 98°C with subsequent melting curve creation by temperature reduction to 80°C (ramp rate 0.05°C/s). Optimal reaction temperatures for the *rplD* primer mixes were selected by means of the shortest detection times achieved. Therefore, mean values (*M*) of detection times and corresponding standard deviation (*SD*) were determined for each tested reaction temperature-primer mix combination. Subsequently, 10-fold serially diluted DNA of *C. jejuni* NCTC 12660 (10 fg/μl–10 ng/μl) was tested with both standard and concentrated primer mixes using the previously specified reaction temperatures to select the most sensitive *rplD* primer mix version. One reaction with 5 μl nuclease-free water instead of DNA template served as a negative template control in each run. Specific melting temperatures of *rplD* LAMP products were determined according to the selected reaction temperature-primer mix combination. The reference range was defined as average melting temperature ± 1°C from three independent measurements during temperature optimization. Individual LAMP assays were initially performed using *cdtC* and *gyrA* primers. Since the intended one-step differentiation of *C. jejuni* and *C. coli* required a combination of these two primer sets, selecting the most appropriate reaction temperature and primer mix versions was based on the development of common reaction kinetics. Mean values of detection times and corresponding standard deviations were calculated as outlined above. In the following, the selected *cdtC* and *gyrA* primer concentrations were merged in one primer mix and tested at their common optimal reaction temperature using 10-fold serially diluted DNA from *C. jejuni* NCTC 12660 and *C. coli* NCTC 12668 (10 fg/μl–10 ng/μl). In each run, one negative template control reaction was carried out as previously described. DNA-based analytical sensitivity and the obtained melting curves were evaluated. The specific melting temperatures for *cdtC* and *gyrA* LAMP products were determined as described for *rplD* LAMP products. All experiments were performed in triplicate. The following LAMP runs included one negative template control reaction as well as one (*rplD* LAMP) or two (*cdtC*–*gyrA* LAMP) positive control reactions using DNA templates of *C. jejuni* NCTC 12660 or both *C. jejuni* NCTC 12660 and *C. coli* NCTC 12668.

### DNA Extraction From Bacterial Target and Non-target Strains

DNA extraction was performed from a total of 174 bacterial strains using the DNeasy Blood and Tissue Kit (Qiagen GmbH). For this purpose, species *C. jejuni* and *C. coli* were cultured on Columbia agar with sheep blood (COLS) (Oxoid Deutschland GmbH, Wesel, Germany) at 42°C for 24–48 h under microaerobic conditions (85% N_2_, 10% CO_2_, and 5% O_2_). Differing from this, other *Campylobacter* spp. as well as *Arcobacter* spp. and *Helicobacter pylori* were cultured at 37°C for 48–72 h. Cultivation of *Bacillus* spp. and remaining strains took place under aerobic conditions for 24 h at 37°C in 10 ml of brain heart infusion (BHI) broth (Oxoid Deutschland GmbH) and on COLS agar, respectively. In the following, DNA was extracted from five to 10 bacterial colonies of each agar plate culture or from 1 ml of enriched BHI broth in accordance with the instruction manual of the DNA extraction kit. Subsequently, DNA purity and concentration of the obtained eluates were analyzed using the spectrophotometer NanoDrop 2000c (Thermo Fisher Scientific GmbH, Dreieich, Germany). The DNA concentration was adjusted to 0.1 ng/μl before templates were used for analytical specificity testing.

### Analytical Specificity

A total of 174 bacterial strains were used to verify the analytical specificity of the LAMP assays (Table 2). Inclusivity testing covered 62 *C. jejuni* and 27 *C. coli* strains, whereas exclusivity was determined by means of 85 other *Campylobacter* and non-*Campylobacter* strains. Non-target species were selected for their close genetic relationship to *C. jejuni* and *C. coli* or because they are present in the same environment or grow under the same conditions as the target species. Melting temperatures of each LAMP product were recorded to verify reaction specificity. All test strains were confirmed via matrix-assisted laser desorption/ionization time-of-flight (MALDI-TOF) analysis using the mass spectrometer microflex^TM^ LT/SH (Bruker Daltonik GmbH, Bremen, Germany) in accordance with the manufacturer’s instructions. *Listeria* spp., *Bacillus* spp., and *Staphylococcus aureus* were pretreated by ethanol/formic acid extraction before spotting, while the other test strains were directly transferred to the MALDI-TOF target plates.

**TABLE 2:** Analytical specificity of the *rplD* and *cdtC*–*gyrA* LAMP assays against target and non-target bacterial strains.

Strain	No. of strains	LAMP	
		*rplD*	*cdtC*–*gyrA*
***Campylobacter* target species**			
*Campylobacter coli* (incl. DSM 4689^*T*^, NCTC 12666, NCTC 12667, NCTC 12668)	27	+	+ (*gyrA*)
*Campylobacter jejuni* (incl. DSM 4688^*T*^, NCTC 12659, NCTC 12660, NCTC 12661, NCTC 12662, NCTC 12663, NCTC 12664, NCTC 12665)	62	+	+ (*cdtC*)
**Non-target species**			
*Aeromonas hydrophila* (DSM 30187^*T*^)	1	−	−
*Arcobacter butzleri* (DSM 8739^*T*^)	1	−	−
*Arcobacter cryaerophilus* (DSM 7289^*T*^)	1	−	−
*Arcobacter skirrowii* (DSM 7302^*T*^)	1	−	−
*Bacillus cereus* (incl. ATCC 11779)	7	−	−
*Bacillus licheniformis*	1	−	−
*Bacillus mycoides* (CCUG 26678^*T*^)	1	−	−
*Bacillus subtilis* (incl. DSM 347)	2	−	−
*Bacillus thuringiensis* (incl. CCUG 7429^*T*^)	3	−	−
*Bacteroides fragilis* (CCUG 4856^*T*^)	1	−	−
*Brochothrix thermosphacta* (CCUG 35132^*T*^)	1	−	−
*Campylobacter fetus* subsp. *fetus* (CCUG 50940)	1	−	−
*Campylobacter fetus* subsp. *venerealis* (CCUG 33899^*T*^)	1	−	−
*Campylobacter helveticus* (CCUG 34042, CCUG 34092)	2	−	−
*Campylobacter hyointestinalis* (CCUG 14169^*T*^)	1	−	−
*Campylobacter lari* (CCUG 29405, CCUG 29406)	2	−	−
*Campylobacter upsaliensis* (CCUG 74242)	1	−	−
*Citrobacter braakii*	1	−	−
*Citrobacter freundii*	2	−	−
*Citrobacter koseri* (DSM 4595^*T*^)	1	−	−
*Citrobacter youngae*	1	−	−
*Enterobacter cloacae* (NCTC 13464)	1	−	−
*Enterococcus faecalis* (DSM 13591, NCTC 8727)	2	−	−
*Enterococcus faecium* (DSM 25389, DSM 25390)	2	−	−
*Escherichia coli* (DSM 1103, DSM 22311, DSM 22316, DSM 22665)	4	−	−
*Hafnia alvei* (DSM 30163^*T*^)	1	−	−
*Helicobacter pylori* (CCUG 47164)	1	−	−
*Klebsiella oxytoca*	1	−	−
*Klebsiella pneumoniae* (NCTC 13465)	1	−	−
*Lactobacillus casei* (DSM 20011^*T*^)	1	−	−
*Lactococcus lactis* (DSM 20481^*T*^)	1	−	−
*Listeria grayi* (DSM 20596)	1	−	−
*Listeria innocua* (DSM 20649^*T*^)	1	−	−
*Listeria ivanovii* (DSM 12491^*T*^)	1	−	−
*Listeria monocytogenes* (incl. DSM 19094)	8	−	−
*Micrococcus luteus* (DSM 1790)	1	−	−
*Morganella morganii*	1	−	−
*Proteus mirabilis* (DSM 4479^*T*^)	1	−	−
*Pseudomonas aeruginosa* (DSM 939)	1	−	−
*Salmonella* Enteritidis (incl. DSM 14221)	5	−	−
*Salmonella* Infantis	1	−	−
*Salmonella* Newport	2	−	−
*Salmonella* Typhimurium (incl. DSM 19587)	5	−	−
*Serratia liquefaciens*	1	−	−
*Shigella flexneri* (DSM 4782^*T*^)	1	−	−
*Shigella sonnei* (DSM 5570^*T*^)	1	−	−
*Staphylococcus aureus* (DSM 18597, DSM 799)	2	−	−
*Streptococcus thermophilus* (CCUG 21957^*T*^)	1	−	−
*Vibrio parahaemolyticus* (DSM 10027^*T*^)	1	−	−
*Yersinia enterocolitica* (DSM 11502)	1	−	−
*Yersinia pseudotuberculosis* (DSM 8992^*T*^)	1	−	−

*ATCC, American Type Culture Collection; CCUG, Culture Collection University of Gothenburg; DSM, German Collection of Microorganisms and Cell Cultures; NCTC, National Collection of Type Cultures; superscript T, type strain.*

### Bacterial Cell-Based Detection Limits of the LAMP Assays

DNA templates obtained from a dilution series of *C. jejuni* NCTC 12660 and *C. coli* NCTC 12668 were used to determine the bacterial cell-based detection limits of the LAMP assays. For this purpose, bacterial cultures were grown on COLS agar for 24 h at 42°C under microaerobic conditions. Subsequently, colonies of the two strains were each suspended in 5 ml of phosphate-buffered saline (PBS) (Carl Roth GmbH und Co. KG, Karlsruhe, Germany) till turbidity of 2 McFarland units (MFU) was measured using a densitometer (Grant Instruments Ltd., Cambridgeshire, United Kingdom). In the following, both cell suspensions were 10-fold serially diluted in Preston broth (Oxoid Deutschland GmbH). Viable cell counts were verified by plating out 100 μl from appropriate dilutions on COLS agar in duplicate. After 48-h incubation at 42°C under microaerobic conditions, plates that showed between 10 and 300 colonies were used for cell counting. Cell concentrations in the initial cell suspensions were calculated as the weighted mean from two successive dilutions. For DNA extraction from the dilution series, a boiling method, as it offers a simple and cost-effective alternative toward the use of an DNA isolation kit, was performed in accordance with the instructions of the German official method L 06.32-1:2013-08 (Bundesamt für Verbraucherschutz und Lebensmittelsicherheit, 2013). Briefly, 1 ml of each dilution was transferred into a 1.5-ml reaction tube and centrifuged for 5 min at 10,000 *g*. After supernatants had been carefully decanted, the cell pellets were washed in 500 μl of PBS. Again, the cell suspensions were centrifuged for 5 min at 10,000 *g*, and supernatants were carefully removed. Cell pellets were resuspended in 300 μl of TE buffer (1 mM of Tris and 0.1 mM of EDTA, pH 8) (Alfa Aesar, Fisher Scientific GmbH, Schwerte, Germany) and then thermally disrupted for 15 min at 95°C. After a 3-min centrifugation step at 14,000 *g* and 4°C, supernatants were transferred into new 1.5-ml reaction tubes and immediately subjected to LAMP analysis. The experiments were performed in triplicate.

### Reference Polymerase Chain Reaction and Culture Methods for the Examination of Meat Samples

Meat samples used for LAMP analysis in this study were comparatively tested for the presence of *C. jejuni* and *C. coli* by multiplex real-time PCR in accordance with the German official method L 06.32-1:2013-08 (Bundesamt für Verbraucherschutz und Lebensmittelsicherheit, 2013) and the standard culture method in accordance with DIN EN ISO 10272-1:2017-09 (International Organization for Standardization, 2017). Primer and probe sequences as well as the composition of reaction mixtures and reaction conditions used for real-time PCR are available in the Supplementary Data. Reactions were carried out using the LightCycler^®^96 instrument (Roche Diagnostics GmbH, Mannheim, Germany). FastStart Essential Probes Master (Roche Diagnostics GmbH) served as master mix in each reaction mixture. For real-time PCR as well as cultural examination, 10 g of each food matrix was homogenized with 90 ml of Preston broth for 2 min at 230 rpm by a stomacher (Seward Ltd., Worthing, West Sussex, United Kingdom). After 24- and 48-h enrichment at 42°C under microaerobic conditions, 1 ml of enrichment liquid was subjected to DNA extraction for real-time PCR analysis. A further 10 μl was spread out on modified charcoal-cefoperazone-deoxycholate agar (mCCDA) (Oxoid Deutschland GmbH). Considering different morphologies, shiny metallic colonies that appeared within 48 h of microaerobic incubation at 42°C were subcultured on COLS agar for 24–48 h. All cultures were archived at −80°C and confirmed at species level by MALDI-TOF analysis as described in Section “Analytical *S*pecificity.”

### Detectability of *Campylobacter jejuni* and *Campylobacter coli* in Artificially Contaminated Minced Meat After Stress Exposure and Subsequent Enrichment

The detection limit of the *rplD* LAMP assay for initial contamination with stressed *C. jejuni* and *C. coli* after enrichment in artificially contaminated minced meat samples was determined. Before inoculation, sample units were tested for the absence of *C. jejuni* and *C. coli* by means of the standard culture method. Artificial contamination of minced meat included two reference strains (*C. jejuni* NCTC 12660 and *C. coli* NCTC 12668) and two field isolates (*C. jejuni* LVL 8 and *C. coli* LVL 22). As described in Section “Bacterial Cell-Based Detection Limits of the LAMP Assays”, cell suspensions of each bacterial strain (turbidity = 2 MFU) were prepared and 10-fold serially diluted in Preston broth. Viable cell counts were confirmed by the plating method as outlined in that Section as well. Per bacterial strain, four 10-g portions of minced meat were spiked with 0–1, 1–10, 10–100, and 100–1,000 CFU using 1 ml from appropriate dilutions. Moreover, four minced meat samples remained non-inoculated and served as negative extraction controls. In the following, all samples underwent storage for 24 h at 4°C in a candle jar to stress bacterial cells at medium level. The candle jar provided slightly reduced oxygen and increased carbon dioxide compared with the air atmosphere, which would have been too detrimental to the survival of *Campylobacter* spp. for the purposes of this experiment (Phebus et al., 1991). After cold storage, the samples were homogenized with 90 ml of Preston broth for 2 min at 230 rpm using a stomacher, followed by microaerobic enrichment for 24 and 48 h at 42°C. At the end of each incubation period, DNA was extracted from 1 ml of enrichment liquid of each sample as outlined in Section “Bacterial Cell-Based Detection Limits of the LAMP Assays”. While cultural examination of samples was continued as described in Section “Reference Polymerase Chain Reaction and Culture Methods for the Examination of Food Samples”, DNA templates were subjected to *rplD* LAMP as well as to real-time PCR analysis. Additionally, *Campylobacter* strains reisolated during cultural examination underwent DNA extraction. Therefore, one colony was picked from COLS agar and directly suspended in 500 μl of PBS. The rest of the extraction procedure complied with the requirements of the German official method L 06.32-1:2013-08. DNA templates obtained from single colonies underwent analysis by *cdtC*–*gyrA* LAMP. Melting temperatures of the obtained LAMP products were recorded and assigned to species *C. jejuni* and *C. coli*. Independent experiments were performed three times in three successive weeks. The detection limit of the *rplD* LAMP assay for initial contaminations with stressed *C. jejuni* and *C. coli* in artificially contaminated samples was defined as the lowest inoculation level that was detectable after sample enrichment in all three repetitions, with results being congruent to the findings of at least one reference method.

### LAMP Assay Validation by Means of Naturally Contaminated Meat Samples

For LAMP assay validation, a total of 101 fresh meat samples including chicken, turkey, beef, and pork were purchased from local grocery stores and investigated for the presence of *C. jejuni* and *C. coli*. *RplD* LAMP results were compared with the findings of real-time PCR and the standard culture method. All samples were wrapped in polyethylene packaging containing protective gas. Sample enrichment, DNA extraction, and the following investigation procedures were performed as described in Section “Detectability of *Campylobacter jejuni* and *Campylobacter coli* in Artificially Contaminated Minced Meat After Stress Exposure and Subsequent Enrichment”. *CdtC-gyrA* LAMP was applied for species differentiation of isolates obtained during cultural examination of the test samples.

### Data Processing and Statistical Analysis

Raw data obtained by LAMP and real-time PCR were processed using the software Genie Explorer (OptiGene Ltd.) available at http://www.optigene.co.uk/support/ and the LightCycler^®^96 application software (Roche Diagnostics GmbH), respectively. Statistical analysis was performed using Microsoft Office Excel (Microsoft Corporation, Redmond, WA, United States). The established *rplD* LAMP assay was evaluated by determining its diagnostic quality criteria with reference to the results of real-time PCR and the standard culture method. This included calculation of diagnostic sensitivity (SE), specificity (SP), and accuracy (AC) as well as positive (PPV) and negative (NPV) predictive values (Garrido-Maestu et al., 2017). Agreement of results was stated as true positive (TP) or true negative (TN). Disagreement of results was stated as false positive (FP) or false negative (FN). The following formulas were used for calculating the previously mentioned parameters:

SE = [TP/(TP + FN)] × 100

SP = [TN/(TN + FP)] × 100

AC = [(TP + TN)/N] + 100 (where N = total number of analyzed samples)

PPV = [TP/(TP + FP)] × 100

NPV = [TN/(TN + FN)] × 100

## Results

### LAMP Assay Optimization

**FIGURE 1:**
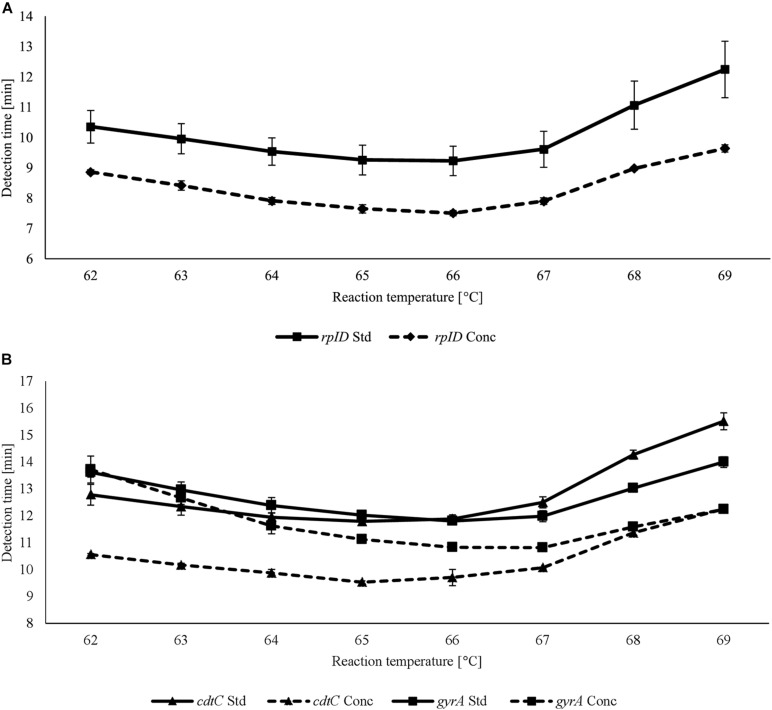
Detection times achieved using standard (Std) and concentrated (Conc) *rplD* **(A)** and *cdtC* and *gyrA* **(B)** primer mixes at different reaction temperatures. Error bars represent the standard deviation of three independent measurements.

**FIGURE 2:**
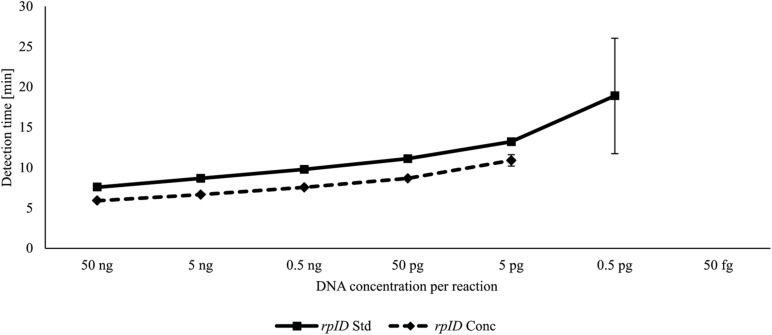
DNA-based analytical sensitivity of the *rplD* LAMP assay using standard and concentrated primer mixes at the optimal reaction temperature of 66°C. Error bars represent the standard deviation of three independent measurements.

**FIGURE 3:**
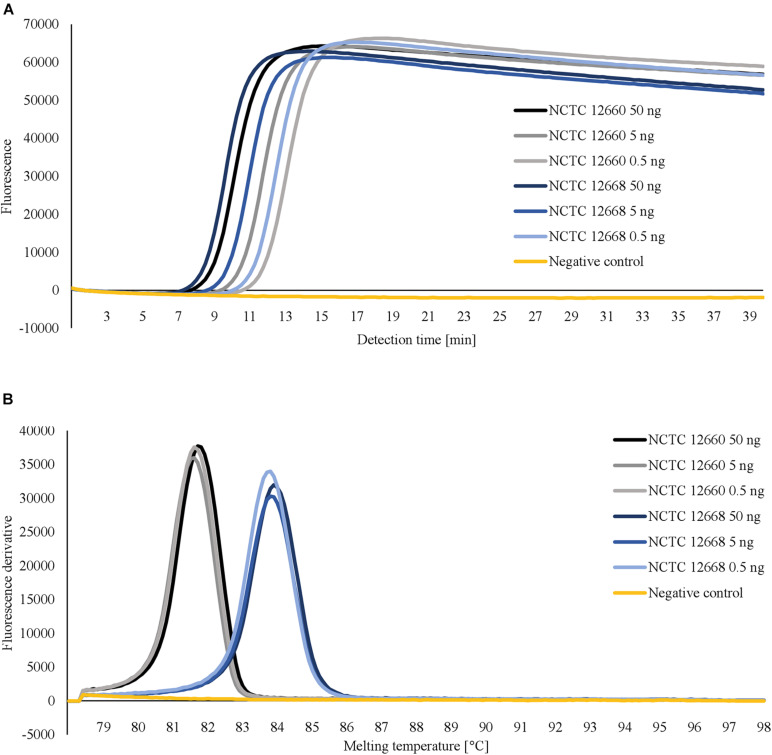
Amplification curves **(A)** and corresponding melting curves of LAMP products **(B)** obtained after testing 10-fold serially diluted DNA templates of *C. jejuni* NCTC 12660 and *C. coli* NCTC 12668 with merged *cdtC*–*gyrA* primers. For a better overview, only the first three dilution levels are shown. Pure cultures of *C. jejuni* und *C. coli* were distinguishable in one reaction by different melting temperatures of their LAMP products.

For each LAMP assay, reaction temperatures and primer concentrations were adjusted to gain optimal reaction conditions. With the use of *rplD* primers, the shortest detection times were achieved at 66°C for both standard and concentrated primer mixes (Figure 1A). Reactions at 66°C were 10 times more sensitive when the standard primer mix was applied (analytical sensitivity = 0.5 pg DNA per reaction) (Figure 2). Thus, subsequent *rplD* LAMP reactions were performed at 66°C using the standard primer mix. Specific melting temperatures of *rplD* LAMP products were defined as 84.9°C ± 1°C. The standard *cdtC* and *gyrA* primer mixes showed common reaction kinetics at 66°C, with mean detection times of 11:53 min:s (*SD* = 00:09 min:s) and 11:48 min:s (*SD* = 00:08 min:s), respectively. Similar reaction kinetics were also observable for concentrated *cdtC* and *gyrA* primer mixes at 69°C, but with longer detection times and far from the 65°C temperature optimum of the DNA polymerase contained in the GspSSD isothermal master mix (ISO-001) (Figure 1B). Thus, standard concentrations of *cdtC* and *gyrA* primers were merged into one primer mix and applied at 66°C in all subsequent LAMP runs. Analytical sensitivity of the combined *cdtC*–*gyrA* primer mix was 5 pg DNA per reaction mixture for both *Campylobacter* species. After amplification (Figure 3A), species differentiation of pure cultures was directly possible by observation of melting curves on the LAMP device (Figure 3B). Melting temperatures of *C. coli*-specific *gyrA* LAMP products (83.9°C ± 1°C) were clearly distinguishable from those of *C. jejuni*-specific *cdtC* LAMP products (81.7°C ± 1°C). In the subsequent *cdtC*–*gyrA* LAMP runs, melting temperatures were determined within descending temperatures from 98 to 78°C for better presentation of the melting curves.

### Analytical Specificity

The analytical specificity of the LAMP assays was tested on 62 *C. jejuni* and 27 *C. coli* as well as 85 other *Campylobacter* and non-*Campylobacter* strains. DNA from all target species was amplified by *rplD* primers, while no amplification signal occurred in any of the non-target strains. Melting temperatures of *rplD* LAMP products ranged between 84.2 and 85.2°C and therefore were specific. *CdtC*–*gyrA* primers induced amplification of each *C. jejuni* and *C. coli* DNA template, with corresponding LAMP products showing species-specific melting temperatures between 80.8–82.1°C and 83.7–84.3°C, respectively. No false-positive signal occurred in any of the tested non-target strains. Thus, both the *rplD* and *cdtC*–*gyrA* LAMP assays showed 100% analytical specificity with respect to the strains tested.

### Bacterial Cell-Based Detection Limits of the LAMP Assays

Bacterial cell-based detection limits of the LAMP assays were determined by means of dilution series from *C. jejuni* and *C. coli*. When the *rplD* LAMP assay was applied, the detection limit for *C. jejuni* and *C. coli* was 5.4 × 10^2^ CFU/ml on average. This corresponded to an analytical sensitivity of 9.0 CFU per reaction. The *cdtC*–*gyrA* LAMP assay detected *C. jejuni* and *C. coli* up to average concentrations of 4.2 × 10^3^ and 6.7 × 10^1^ CFU/ml, respectively, corresponding to an analytical sensitivity of 70.0 and 1.1 CFU per reaction, respectively.

### Detectability of *Campylobacter jejuni* and *Campylobacter coli* in Artificially Contaminated Minced Meat After Stress Exposure and Subsequent Enrichment

**FIGURE 4:**
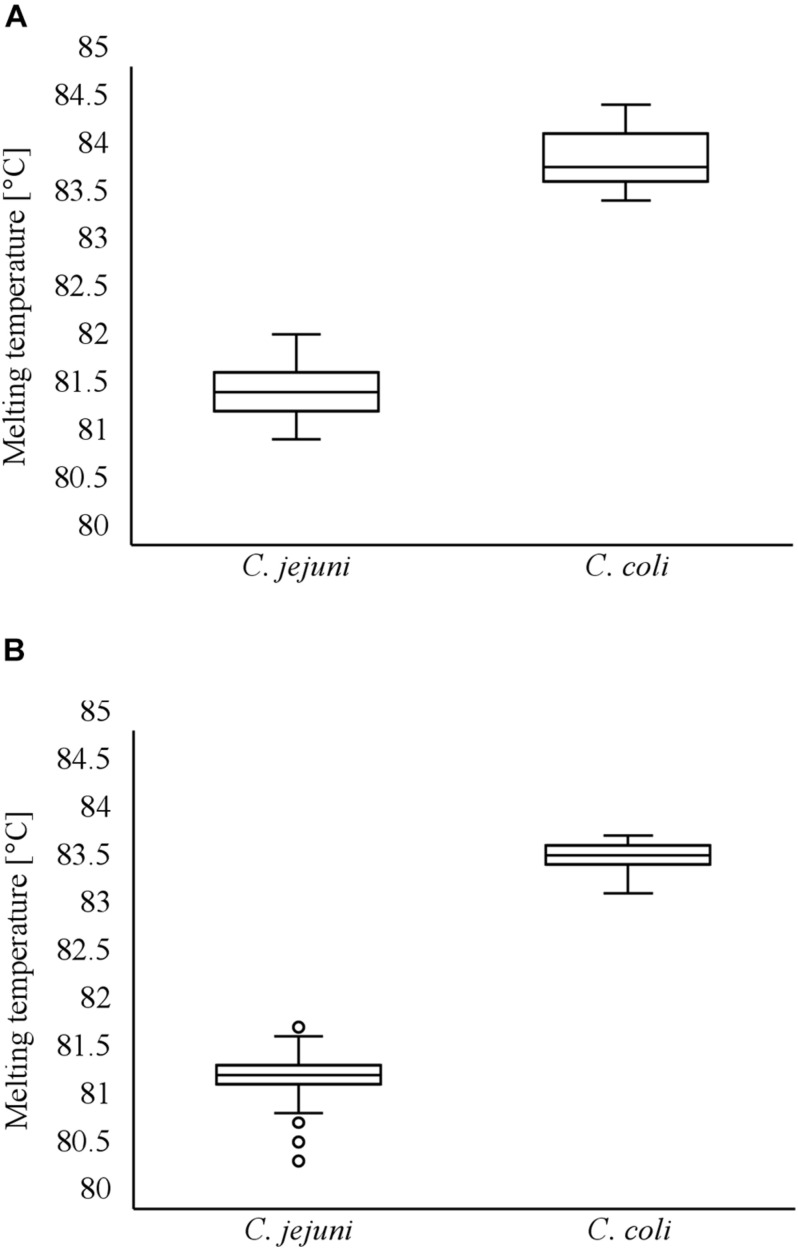
Distribution of melting temperatures of LAMP products during investigation of artificially contaminated minced meat using *cdtC*–*gyrA* LAMP **(A)**. Distribution of melting temperatures of LAMP products during investigation of naturally contaminated meat samples using *cdtC*–*gyrA* LAMP **(B)**.

Minced meat samples with varying degrees of artificial contamination were comparatively examined for the presence of *C. jejuni* and *C. coli* by *rplD* LAMP, real-time PCR, and the standard culture method. With the use of *rplD* LAMP, initial inoculation levels of 10–100 CFU/g resulted in a detection rate of 100% after 24-h incubation, whereas the detection limit improved to initial contaminations of 1–10 CFU/g when samples were enriched for 48 h. All samples that were not artificially contaminated before incubation revealed negative *rplD* LAMP results. In most cases, *rplD* LAMP showed positive findings in initially lower inoculated samples. Nonetheless, strains used for artificial contamination could not be re-enriched at each inoculation level in each trial. The total numbers of positive and negative test results obtained by *rplD* LAMP and reference methods are shown in Table 3, reflecting the detection competence of *rplD* LAMP at initial inoculation levels below 10–100 or 1–10 CFU/g. Irrespective of the underlying reference method, the *rplD* LAMP assay correctly identified 93.3% of the samples after 24-h enrichment. Compared with real-time PCR, *rplD* LAMP showed two false-positive as well as two false-negative results. Differing from this, results were false positive and false negative in one and three cases, respectively, with reference to the standard culture method. When samples were incubated for 48 h, agreement of all three test methods was 100%. Interestingly, samples that tested false positive with LAMP after 24-h enrichment were found to be positive by all examination methods using 48-h sample incubation. Melting temperatures of all *rplD* LAMP products were specific. *C. jejuni* and *C. coli* strains that were recovered during cultural examination of the artificially contaminated minced meat samples were 100% identifiable and distinguishable using the *cdtC*–*gyrA* LAMP assay with respective amplification products showing species-specific melting temperatures (Figure 4A).

**TABLE 3:** Total numbers of positive (+) and negative (−) results obtained by *rplD* LAMP, real-time PCR, and the standard culture method, testing artificially contaminated minced meat samples at four inoculation levels after 24- and 48-h enrichment.

	0–1 CFU		1–10 CFU		10–100 CFU		100–1000 CFU	
	+	−	+	−	+	−	+	−
24-h enrichment								
*rplD* LAMP	1	11	5	7	10	2	12	0
Real-time PCR	1	11	7	5	9	3	11	1
Standard culture method	1	11	7	5	10	2	12	0
48-h enrichment								
*rplD* LAMP	2	10	8	4	12	0	12	0
Real-time PCR	2	10	8	4	12	0	12	0
Standard culture method	2	10	8	4	12	0	12	0

### Diagnostic Efficiency of the LAMP Assays in Natively Contaminated Meat Samples

The LAMP assays were validated by investigating 101 natively contaminated meat samples for the presence of *C. jejuni* and *C. coli*. Overall, 43.6, 48.5, and 47.5% of the samples revealed positive findings by *rplD* LAMP, real-time PCR, and the standard culture method after 24-h enrichment, respectively. When samples were incubated for a further 24 h, the proportion of positive findings increased to 52.5% by *rplD* LAMP as well as by the reference methods. After 24-h enrichment, the diagnostic accuracy of *rplD* LAMP was 95.0 and 94.1% according to the results of real-time PCR and cultural examination, respectively. With reference to real-time PCR, five samples were tested false negative by *rplD* LAMP, whereas one false-positive and five false-negative results were retrieved in comparison with the standard culture method. The resulting diagnostic quality criteria are shown in Table 4. When samples were incubated for 48 h, the diagnostic accuracy of the *rplD* LAMP assay measured against both reference methods increased to 100%. As described in Section “Detectability of *Campylobacter jejuni* and *Campylobacter coli* in Artificially Contaminated Minced Meat After Stress Exposure and Subsequent Enrichment”, samples tested false positive by LAMP after 24-h enrichment gained positive results by all examination methods after incubation for a further 24 h. All *rplD* LAMP products showed specific melting temperatures. A total of 119 *C. jejuni* and 40 *C. coli* strains were isolated during cultural examination and were correctly identified using the *cdtC*–*gyrA* LAMP assay. Except in two cases, all *cdtC* and *gyrA* LAMP products revealed specific melting temperatures that enabled species differentiation (Figure 4B). Two *cdtC* LAMP products showed melting temperatures of 80.5 and 80.6°C. These slightly deviated from the reference range but still allowed clear assignment of the species.

**TABLE 4:** Diagnostic criteria of the *rplD* LAMP assay compared with real-time PCR and the standard culture method after examination of naturally contaminated meat products after 24- and 48-h incubation.

Criteria	LAMP real-time PCR		LAMP (standard culture method)	
	24 h	48 h	24 h	48 h
SE (%)	89.8	100	89.6	100
SP (%)	100	100	98.1	100
AC (%)	95.0	100	94.1	100
PPV (%)	100	100	97.7	100
NPV (%)	91.2	100	91.2	100

*SE, diagnostic sensitivity; SP, diagnostic specificity; AC, diagnostic accuracy; PPV, positive predictive value; NPV, negative predictive value.*

## Discussion

In this study, *rplD*, *cdtC*, and *gyrA* gene-based LAMP assays were combined to enable rapid detection of *C. jejuni* and *C. coli* in meat products followed by one-step species differentiation on agar plates. Both the *rplD* and *cdtC*–*gyrA* LAMP assays achieved 100% inclusivity of target species and exclusivity of non-target species throughout the entire experiments. Except for the *cdtC* gene, selected genes have not been previously used for nucleic acid amplification-based detection of *C. jejuni* and *C. coli*. The *rplD* gene encodes the L4 ribosomal protein, which is probably involved in the formation of the polypeptide exit tunnel in 70S bacteria (Franceschi et al., 2004). Mutations within this gene region have been linked to the emergence of macrolide resistance (Cagliero et al., 2006). However, *rplD*-based LAMP detection was not affected, since BLAST analysis revealed no relevant polymorphisms within the primer sequences. This target region offers the advantage of selective simultaneous detection of the most relevant *Campylobacter* species *C. jejuni* and *C. coli* using a single primer set. In other studies, either different primer sets were used or the detection spectrum extended to a less selective detection of all thermotolerant *Campylobacter* based on the 16S RNA gene (Yamazaki et al., 2008; Romero et al., 2016). Like the *rplD* gene, the *gyrA* gene is primarily known for its mutations causing changes in the encoded subunit of the enzyme DNA gyrase and therefore being involved in the mediation of fluoroquinolone resistance (Wieczorek and Osek, 2013). *GyrA* LAMP primer sequences did not cover relevant polymorphisms as well. Due to the importance of genes *rplD* and *gyrA* for ribosomal function and nucleotide metabolism, it can be assumed that their nucleotide sequences are constantly present in the *Campylobacter* genome and therefore constitute suitable targets for LAMP detection. As a part of the *cdt* operon, the expression of the *cdtC* gene is involved in the formation of cytolethal distending toxin representing a well-researched virulence factor of *Campylobacter* spp. (Smith and Bayles, 2006). Several studies determined the prevalence of this gene by PCR-based investigation of *C. jejuni* isolates and showed that the sequence was not amplifiable in each strain (Krutkiewicz and Klimuszko, 2010; Findik et al., 2011). In contrast, the *cdtC* sequence could be amplified in each of the tested *C. jejuni* strains using *cdtC*–*gyrA* LAMP. Besides the randomness of this finding, it is also possible that the prevalence of the *cdtC* gene among *C. jejuni* isolates was underestimated in previous research due to limited analytical specificity of the PCR assays used. Since LAMP only occurs when the four basic primers (F3, B3, FIP, and BIP) bind to six distinct regions within the target genes, it provides a high degree of analytical specificity (Mori and Notomi, 2009), which, in the present study, might exceed that of PCR.

For the first time in a LAMP study, detection limits for *C. jejuni* and *C. coli* were determined on the basis of enriched artificially contaminated meat samples, taking into account various cell stressors and their influence on growth behavior of *Campylobacter*. This enabled realistic assessment of assay performance and confirmed the suitability of *rplD* LAMP for application in meat products. A similar approach was recently undertaken by Babu et al. (2020), who investigated spinach artificially inoculated with various *Campylobacter* spp. Spiked samples were first stored at 4°C for 1 day and then rinsed with 75 ml of Bolton broth. After rinses had been enriched for 24 h, absolute initial contamination levels of 1–5 CFU were detectable by LAMP and a culture method. In the present study, the lowest initial contamination level found to be 100% detectable after 24-h sample incubation was 100–1,000 CFU. However, target species could also be detected in lower inoculated samples, but unlike the study by Babu et al. (2020), re-enrichment of strains was not possible in all cases. Nonetheless, these studies are difficult to compare since they differ substantially in both the food matrices investigated and the methods used. Additionally, the LAMP assay established by Babu et al. (2020) has not been validated by application in natively contaminated food samples. A different approach for determining the limit of detection was demonstrated by Yamazaki et al. (2009), who used artificially inoculated enrichment broth previously collected from *Campylobacter*-free chicken samples. This procedure does not take into account *Campylobacter* fitness and its relevance for detection probability after enrichment. The results of the present study demonstrated that the condition of *Campylobacter* cells strongly influences enrichment ability and should therefore be considered for appropriate evaluation of assay limitation.

Assay validation revealed that the probability of a culture-positive native sample being correctly identified by the *rplD* LAMP assay was approximately 90% after 24-h enrichment using boiling extraction of DNA. Although the average analytical sensitivity of 9.0 CFU per reaction obtained by *rplD* LAMP was roughly comparable with that determined by Yamazaki et al. (2009), their LAMP assay showed less (76.2%) or higher (100%) diagnostic sensitivity after 24–48 h of sample incubation depending on the use of a commercially available kit or a complex three-step-centrifugation procedure for DNA extraction. Dong et al. (2014) developed a *C. jejuni*-specific LAMP assay that focused on investigating cattle farm samples after overnight enrichment. Detection probability for culture positive samples by LAMP only reached 84.4%, although a similar boiling extraction of DNA was performed. The comparison with these studies highlights the robustness of *rplD* LAMP, coping with simple DNA extraction and still achieving high detection rates among positive samples. Referring to diagnostic specificity, the *rplD* LAMP assay exceeded both the 91.5% level determined by Dong et al. (2014) and that of 97.4% determined by Yamazaki et al. (2009). Comparative values for diagnostic quality criteria of *C. jejuni*- and *C. coli*-detecting LAMP assays measured against reference PCR assays are largely missing in the literature. Zang et al. (2017) found high result agreement between *C. jejuni*-specific LAMP and PCR during investigations of food samples, with diagnostic sensitivity, specificity, and accuracy of LAMP being 100, 98.1, and 98.4%, respectively. However, there were two major differences from the present study that limit comparability of the results. On the one hand, Zang et al. (2017) directly detected *Campylobacter* from sample rinses; on the other hand, their PCR assay was only established during the study and therefore lacks comprehensive validation. High result agreement between LAMP and PCR was also reported by Quyen et al. (2019). Diagnostic sensitivity, specificity, and accuracy were 100, 97.9, and 98.4%, respectively, for the detection of *C. jejuni* and *C. coli* in chicken feces. Nonetheless, assays were not performed on templates from meat samples potentially containing amplification inhibitors due to prior enrichment in Preston broth (Yamazaki et al., 2009). As with LAMP, a closer look at the reference real-time PCR used in the present study and other comparable PCR assays reveals that detection results for *C. jejuni* and *C. coli* are usually not completely consistent with findings of the standard culture method (Mateo et al., 2005; Mayr et al., 2010). Nucleic acid amplification-based detection occasionally shows higher sensitivity than cultural investigation procedures and therefore can provide false-positive results (Yang et al., 2003; Zang et al., 2017). This was also observable in the present study when false-positive findings by *rplD* LAMP after 24-h sample enrichment could be revised after another 24-h incubation period. Moreover, unlike the standard culture method, PCR and LAMP detect non-viable *Campylobacter* as well as *Campylobacter* in their viable but non-culturable state constituting a response to adverse environmental conditions (Jackson et al., 2009). False-positive examination results toward cultural findings might also be due to this characteristic of nucleic acid amplification-based techniques (Lund et al., 2004). However, if sample enrichment is required, diagnostic sensitivity can be impaired by non-culturable *Campylobacter* as well, or by too short incubation periods with the consequence of obtaining false-negative results (Saiyudthong et al., 2015). Heat or cold treatment during processing, gas atmospheres used for packaging, and storage conditions of meat products occur as stressors for *Campylobacter* cells (Duqué et al., 2019). For example, it was shown that cold and oxidative stress results in *Campylobacter* cell reduction and extended lag phases during growth (Lanzl et al., 2020). Failed detection after 24-h incubation of samples therefore might be explained by insufficient multiplication of the target species to cell amounts below the bacterial cell-based detection limit of the *rplD* LAMP assay. However, diagnostic uncertainties, even for low cell numbers as used for artificially contaminated samples, could be eliminated after 48-h enrichment. This qualifies the *rplD* LAMP as a potential official method for detecting *C. jejuni* and *C. coli* in meat products.

Sabike and Yamazaki (2019) recently demonstrated two singleplex LAMP assays that revealed species-specific melting temperatures for *C. jejuni* and *C. coli* LAMP products. In the present study, the principle of melting temperature analysis has been used in the development of a duplex LAMP assay, representing a simple and new approach for one-step species differentiation in pure cultures. *CdtC*–*gyrA* LAMP allowed clear assignment of target species to melting temperatures of 81.7°C ± 1°C (*C. jejuni*) and 83.9°C ± 1°C (*C. coli*). The slight temperature deviation in measurements of 80.5°C and 80.6°C for two *C. jejuni*-LAMP products during native sample testing were most likely caused by an altered target gene sequence in these isolates. Melting temperature analysis has already been used for multiplex detection of other pathogens or integration of internal amplification controls into LAMP reactions without the need for multiple fluorescence channel providing devices (Liu et al., 2017; Romero and Cook, 2018; Sange et al., 2019). Nevertheless, unlike the LAMP products of the artificially created internal amplification control sequences, species-specific amplicon-based LAMP products reveal accidental melting temperatures and must therefore first be selected according to the trial-and-error principle.

As the monitoring of *Campylobacter* mainly refers to primary production and the stage of slaughtering, attention should be given to a possible on-site application of the *rplD* LAMP assay for direct detection of *C. jejuni* and *C. coli* without enrichment culture in frequently highly contaminated feces or carcasses. The present study focused on food inspection, since rapid methods for detecting *Campylobacter* in products at retail level are only sparsely available. Thus, against the background of high infection rates, the established LAMP assay system can significantly contribute to preventive consumer protection.

## Conclusion

The *rplD* and *cdtC*–*gyrA* gene-based LAMP assay system established in this study provides a suitable tool for rapid, sensitive, and specific detection as well as differentiation of *C. jejuni* und *C. coli* in processed meat products. Although *rplD* LAMP enables sample screening after 24 h of incubation, extended sample enrichment of 48 h is recommended to increase diagnostic accuracy to reference method level. Target species are reliably distinguishable by melting temperatures of amplification products using *cdtC*–*gyrA* LAMP. This offers deeper sample analysis in the case of specific questions such as the collection of prevalence data. Even though sample enrichment is necessary for reliable test results, the LAMP assays described in this study are suitable for application in a restricted environment and could therefore be used, for example, in a minimally equipped mobile laboratory. Future investigations might concentrate on optimizing and refining the DNA extraction procedure, without depriving it of the advantages of its simple and cost-effective performance but improving the diagnostic quality criteria of the *rplD* LAMP assay after 24-h enrichment. Prospective validation studies should refer to other risk foods such as milk and dairy products. Following the approach of LAMP product characterization by melting temperature evaluation, designing an internal amplification control might be useful for *rplD* LAMP coping with problematic matrices and enrichment media. Overall, the established LAMP system is promising for a wide range of applications. It could provide an instrument for food monitoring, which can be expected to become more important in the context of prospective systematic control strategies against *Campylobacter*.

## Data Availability Statement

The original contributions presented in the study are included in the article/Supplementary Material, further inquiries can be directed to the corresponding author/s.

## Author Contributions

AK performed the experiments and wrote the manuscript. AK, AB, US, and AA designed the experiments. MA, SK, and US provided resources for the study. MP coordinated the research. AA was responsible for project administration and funding acquisition. All the authors proofread and edited the manuscript.

## Conflict of Interest

The authors declare that the research was conducted in the absence of any commercial or financial relationships that could be construed as a potential conflict of interest.
