# Supplementary material for: Combined Loop-Mediated Isothermal Amplification Assays for Rapid Detection and One-Step Differentiation of Campylobacter jejuni and Campylobacter coli in Meat Products
Source: Front Microbiol. 2021 Jun 9;12:668824. doi: 10.3389/fmicb.2021.668824 (PMC8219907; doi:10.3389/fmicb.2021.668824)
Supplement: Supplementary file 1 [file Data_Sheet_1.pdf]

Supplementary Table 1 Real-time PCR assay used as reference method in this study (in accordance with the official method BVL L 06.32-1:2013-08 published in the Food, Feed and Consumer Goods Code of the Federal Republic of Germany)

**Primers and probes**	
Designation	Sequence
mapA-fw	CTGGTGGTTTTGAAGCAAAGATT
mapA-re	CAATACCAGTGTCTAAAGTGCGTTTAT
mapA-probe	[FAM]-TTGAATTCCAACATCGCTAATGTATAAAAGCCCTTT-[BHQ1]
*gyrA*1-fw1	GATAAAGATACGGTTGATTTTGTACC
*gyrA*1-re1	CAGCTATACCACTTGATCCATTAAG
*gyrA*1-fw2	GATAAAGATACAGTTGATTTTATACC
*gryA*1-re2	TGCAATACCACTTGAACCATTA
*gyrA*1-probe	[ROX]-TTATGATGATTCTATGAGTGAGCCTGATG-[BHQ2]
ceuE-fw	AAGCTCTTATTGTTCTAACCAATTCTAACA
ceuE-re	TCATCCACAGCATTGATTCCTAA
ceuE-probe	[CY5]-TTGGACCTCAATCTCGCTTTGGAATCATT-[BHQ2]
IPC-ntb2-fw	ACCACAATGCCAGAGTGACAAC
IPC-ntb2-re	TACCTGGTCTCCAGCTTTCAGTT
IPC-probe	[HEX]-CACGCGCATGAAGTTAGGGGACCA-[BHQ2]
**Internal amplification control**	
IPC	ACCACAATGCCAGAGTGACAACAGAAGATCACGCGCATGAA-GTTAGGGGACCAGCCGGTCTGTTCTCGTGGGATTTGTTATTTT-CTTTACGAAGTCCATTGAAACTGAAAGCTGGAGACCAGGTA

PCR reaction mixture

Component	Volume/reaction (µl)	Concentration
Master mix	12.5	
mapA-fw	0.375	10 µM
mapA-re	0.375	10 µM
*gyrA*1-fw1	0.375	10 µM
*gyrA*1-re1	0.375	10 µM
*gyrA*1-fw2	0.375	10 µM
*gyrA*1-re2	0.375	10 µM
IPC-ntb2-fw	0.375	10 µM
IPC-ntb2-re	0.375	10 µM
ceuE-fw	0.375	10 µM
ceuE-re	0.375	10 µM
mapA-probe	0.25	25 µM
gyrA-probe	0.25	25 µM
IPC-ntb2-probe	0.25	25 µM
ceuE-probe	0.25	25 µM
H_2O	1.75	
IPC	1	25 copies/µl
DNA template	5	

Assay profile

Step	Temperature	Time
Initial denaturation	95°C	10 min
Three-step amplification (45 cycles)		
Denaturation	94°C	30 s
Annealing	60°C	60 s
Extension	72°C	30 s
